# Quadrivalent HPV (4vHPV) vaccine immunogenicity and safety in women using immunosuppressive drugs due to solid organ transplant

**DOI:** 10.3389/fcimb.2024.1452916

**Published:** 2024-11-04

**Authors:** Karina Takesaki Miyaji, Vanessa Infante, Camila Melo Picone, Joakim Dillner, Hanna Kann, Carina Eklund, José Eduardo Levi, Ana Carolina Soares de Oliveira, Amanda Nazareth Lara, Lyca Suzuki Kawakami, Maricy Tacla, Cristina Paula Castanheira, Philippe Mayaud, Ana Marli Christovam Sartori

**Affiliations:** ^1^ Departamento de Infectologia e Medicina Tropical da Faculdade de Medicina da Universidade de Sao Paulo (FMUSP), Sao Paulo, Brazil; ^2^ Clinica de Molestias Infecciosas e Parasitarias do Hospital das Clinicas da Faculdade de Medicina da Universidade de Sao Paulo (HCFMUSP), Sao Paulo, Brazil; ^3^ Centro de Referencia para Imunobiológicos Especiais, HCFMUSP, Sao Paulo, Brazil; ^4^ Department of Laboratory Medicine, Karolinska Institute, Stockholm, Sweden; ^5^ Department of Microbiology and Immunology, Gothenburg University, Gothenburg, Sweden; ^6^ Laboratório de Investigação Medica – Virologia, Instituto de Medicina Tropical, Sao Paulo, Brazil; ^7^ Faculdade de Medicina, Universidade de Sao Paulo, Sao Paulo, Brazil; ^8^ Clínica de Ginecologia, HC-FMUSP, Sao Paulo, Brazil; ^9^ Clinical Research Department, London School of Tropical Medicine and Hygiene (LSHTM), London, United Kingdom

**Keywords:** papillomavirus vaccines, immunogenicity, safety, immunosuppression, solid organ transplant, cancer prevention

## Abstract

**Introduction:**

Immunocompromised persons are at high risk of persistent Human Papilloma Virus (HPV) infection and associated diseases. Few studies evaluated HPV vaccines in immunocompromised persons. This study aimed to evaluate the quadrivalent HPV vaccine (4vHPV) immunogenicity and safety in solid organ transplant (SOT) recipients, in comparison to immunocompetent women (IC).

**Methods:**

Open-label clinical trial that enrolled SOT recipients and immunocompetent women aged 18 to 45 years. All participants received three doses of 4vHPV vaccine. Blood samples were drawn for evaluation of immune responses at baseline and one month after the third vaccination. Seroconversion rates and antibody geometric mean concentration (GMC) against HPV 6, 11, 16, 18, 31, 35, 52 and 58 were measured with in-house multiplexed serology assay (xMAP technology). Follow-up for the local and systemic adverse events (AEs) continued for seven days after each vaccination. Severe AEs were evaluated throughout the study.

**Results:**

125 SOT and 132 immunocompetent women were enrolled; 105 (84%) SOT and 119 (90%) immunocompetent women completed the study. At baseline, HPV seropositivity was not significantly different between groups. Seroconversion rates were significantly lower in SOT (HPV18, 57%; HPV6 and 16, 69%; and HPV11, 72%) than in immunocompetent women (100% seroconversion to all vaccine types) (*p*<0.001). Antibody GMCs of all four HPV vaccine types were also significantly lower in SOT (*p*<0.001). Pain in the injection site and headache were the most frequent adverse event in both groups. Local pain was more frequent in immunocompetent women than in SOT recipients. Rates of other AEs were comparable in both groups.

**Conclusion:**

4vHPV vaccine was well-tolerated by SOT recipients. We found strong evidence of lower humoral immune responses to 4vHPV vaccine in SOT compared to immunocompetent women, which strengthen recommendation of routine cervical cancer screening in SOT recipients regardless of HPV vaccination status.

## 1 Introduction

Immunocompromised persons such as those living with HIV and solid organ transplant (SOT) recipients are at higher risk of persistent HPV infection and related diseases, such as cancers (Reusser et al., 2015) and genital warts (Gormley and Kovarik, 2012; Wieland et al., 2014). In a previous study, we found prevalence of 29% of any type HPV-DNA and 19.4% of high-risk HPV-DNA in cervical samples of SOT women aged 18 to 45 years. SOT recipients also had a significant higher frequency of high-grade HPV related cervical lesions (5.3%) in comparison to immunocompetent women (0.8%, *p*=0.001) (Miyaji et al., 2022). Another study, in USA, found a 3- to 20-fold higher incidence of *in situ* HPV-related cancers and a 2- to 7-fold higher incidence of invasive HPV-related cancers among SOT recipients in comparison to the general population (Madeleine et al., 2013). These findings strengthen the need to optimize HPV prevention in SOT and other immunocompromised groups.

Primary prevention through HPV vaccination is the most effective strategy to prevent HPV infection and related diseases (Patel et al., 2018; Brotherton, 2019; Drolet et al., 2019). Secondary prevention by screening with a high-performance HPV-DNA test, which offers superior specificity than cytology-based screening, and treatment of cancer precancerous lesions, through cervical conization or electrocoagulation diathermy (World Health Organization, 2020; D’Augè et al., 2024), may prevent up to 80% of cancers.

In Brazil, the quadrivalent HPV vaccine (4vHPV) was introduced in the National Immunization Program (NIP), targeting girls aged 11-14 years, in 2014. Successive expansions in the vaccination target group have been made since then. Currently, 4vHPV is freely available for girls and boys aged 9 to 14 years, in a single dose schedule, and also for women and men living with HIV/AIDS, solid organ and hematopoietic stem cell transplant recipients, patients with cancer and persons using immunosuppressants, up to 45 years of age, in a three-dose schedule (0, 2 and 6 months) (Ministerio da Saúde et al., 2013).

Few studies that evaluated HPV vaccines in immunocompromised individuals have shown lower immunogenicity in persons living with HIV (Toft et al., 2014) and those using immunosuppressive therapy, such as patients with chronic inflammatory diseases and SOT recipients (Jacobson et al., 2013; Mok et al., 2013; Soybilgic et al., 2013; Gomez-Lobo et al., 2014; Nelson et al., 2016; Nailescu et al., 2020; Boey et al., 2021).

This study aimed to evaluate the immunogenicity and safety of 4vHPV vaccine in immunosuppressed solid organ transplanted (SOT) women compared to immunocompetent women, in a single center in Sao Paulo, Southeast of Brazil.

## 2 Materials and methods

### 2.1 Study population

This open-label, non-randomized clinical trial was conducted at the Reference Center for Special Immunobiologicals (CRIE) of the Hospital das Clinicas da Faculdade de Medicina da Universidade de Sao Paulo (HC-FMUSP), from July 12^th^, 2017 to June 23^rd^, 2019.

Women aged 18 to 45 years were recruited in two groups: SOT recipients using immunosuppressive therapy and immunocompetent women. Inclusion criteria for SOT women were: an interval of at least six months after the transplantation (kidney, liver, heart or lung) and using immunosuppressive drugs. Inclusion criteria for the immunocompetent women comparator group were not having any immunocompromising condition and not using immunosuppressive medication. Exclusion criteria for both SOT and immunocompetent groups were: pregnancy or breast-feeding, any other conditions associated to immunocompromise, such as HIV/AIDS, neoplasms or primary immunodeficiencies, chemotherapy or radiotherapy for cancer treatment, within six months previous to study enrollment; use of any immunobiological; and history of HPV vaccination, anogenital warts or any cervical, vulvar, vaginal, or anal lesion related to HPV.

The following data were collected: age, color, educational level, age at menarche and at sexarche, sexual orientation, number of sexual partners, number of pregnancies, contraception use, previous sexually transmitted infections (STIs), known comorbidities, weight and height for body mass index calculation (BMI) and, for SOT women, use of immunosuppressive drugs.

All participants were scheduled to receive three doses of 4vHPV, constituted by recombinant L1 surface protein of HPV types 6, 11, 16 and 18 with aluminum hydroxyphosphate sulfate as adjuvant, produced by Merck / Butantan Institute. The vaccine was administered intramuscularly at deltoid area (with needle length 25 x 0.7mm), at 0, 2, and 6 months. 4vHPV vaccine lots used for routine immunization were administered for the study participants and recommended routine vaccines were allowed to be administered concomitantly.

HIV rapid test was performed for all participants at enrollment. Pregnancy test was done before each vaccine dose. A 5ml venous blood sample was collected at enrolment (prior to the first vaccine dose) and approximately 28 days after the third vaccine dose for HPV serological testing. Whole blood samples were kept at room temperature for no more than 8 hours before they were sent to local laboratory in Sao Paulo where the samples were centrifugated, aliquoted, and stored at -20°C until shipped in a single batch to the laboratory in Stockholm, Sweden, for testing.

### 2.2 Safety evaluation

Immediate local and systemic adverse reactions were evaluated 30 minutes after each vaccine dose for all participants. Systemic and local solicited and unsolicited adverse events (AE) were evaluated within seven days after each vaccination through a diary given to all participants. Solicited AEs included: pain, oedema and erythema at the injection site, fever, headache, myalgia, nausea, vomiting, malaise, diarrhea, rash, wheezing, angioedema, drowsiness and dizziness. Serious adverse events, such as transplanted organ rejection, were monitored during the entire study period, according to the routine of each transplant service. AE intensity was classified according to Food and Drug Administration (FDA) guidelines (Food and Drug Administration et al., 2007).

### 2.3 Laboratory tests

Anti-HPV serology was conducted at the Department of Laboratory Medicine, Karolinska Instituted, in 2019. An in-house multiplexed serology assay (xMAP technology) with mammalian cell-line–derived pseudovirions of HPV6/11/16/18/31/33/52/58, coupled to the heparin-coated polystyrene carriers was employed (Faust et al., 2010; Faust et al., 2013; Artemchuk et al., 2018). The assay performance was rigorously validated using serum samples from women with positive HPV-DNA in cervical sample, confirmed by molecular testing (Faust et al., 2010; Faust et al., 2013; Artemchuk et al., 2018). Average coefficient of variation of our eight-plex assay was 20.7%.

Sera were tested using 50x to 328050x dilutions with a 3-fold increase per step. Samples were classified as seropositive according to the HPV type-specific cut-off values based on reactivity of a negative control serum panel from 99 Brazilian children (average age 5.2 years, range: 1.5-7.4). As per WHO HPV Labnet Manual (World Health Organization (WHO), 2009), cut-off levels were assigned by averaging the median fluorescence intensity (MFI) values of a negative control serum panel plus 3 standard deviations. If the calculated value was less than 400 MFI, an arbitrary level of 400 MFI was assigned. Data on HPV-45 antibody was excluded due to antigen performance failure. Antibody levels of seropositive samples were further translated either into international or into arbitrary units. Anti-HPV16 and HPV18 antibody levels were calculated in international units (IU) by their calibration against WHO International Standard serum (NIBSC codes: 05/134 and 10/140) using reference factor 10 for HPV 16 and 16 for HPV 18 (Ferguson et al., 2011; World Health Organization, 2013). For the HPV types, where international standard sera were not yet established by the time of laboratory testing, we used arbitrary units (AU) defined by a pool of sera from HPV vaccine recipients. Arbitrarily assigned reference factors 1000, 400, 100, 25, 200, and 200 were used for HPV6, 11, 31, 33, 52, and 58, respectively. The parallel line method (PLL) was used to calculate antibody titers relative to the reference (Reizenstein et al., 1995; Grabowska et al., 2002).

Serum samples from different SOT groups and from immunocompetent women were randomized and equally represented on each of the serological plates for laboratory testing, to minimize assay-related variation in the results.

### 2.4 Statistical analysis

The sample size calculation was based on the comparison of HPV seroconversion rates for each of the 4vHPV vaccine types in SOT recipients and immunocompetent women. Based on the results of a previous study involving SOT recipients and immunocompetent women in Canada (Kumar et al., 2013), a sample size of 109 women in each group was deemed sufficient to detect 10% difference of post-vaccination seroconversion rates between the two groups, with a power of 80%, precision of 5% and assuming 10% of dropouts. This study was part of a larger study that also enrolled women with Systemic Lupus Erythematous (SLE). To harmonize the number of participants in all groups, we adopted the larger sample size required for the SLE group, which was 125 in each group.

The electronic capture forms and database were built using REDCap 9.8.5 **©** 2020 (Vanderbilt University, Nashville, USA) and all statistical analyses were conducted using R for Windows, version 3.6.1.

The primary outcome, i.e., immune response to vaccination, was calculated as (i) the proportion of participants who seroconverted for each HPV vaccine type (6, 11, 16 and 18); and (ii) by the geometric mean concentration (GMC) of antibody levels in IU or in AU against the four HPV vaccine types, four weeks after the third vaccine dose. Each HPV type was analyzed separately. Participants who were seropositive for a HPV type at enrolment were excluded from subsequent analyses involving that specific type, but were included in the analysis of other HPV types for which they were negative at enrolment.

An exploratory analysis evaluated cross-type responses to alpha-HPV types additionally targeted by the 9vHPV vaccine (HPV-31, -33, -52, and 58).

Seroconversion rates (from seronegative at enrolment to seropositive post-vaccination) were compared between the two groups of women using the Chi-square test (χ2). GMC of antibody levels IU or AU with 95%CI were calculated. We used the Shapiro-Wilk test to check if antibody GMC followed normal distribution, and since they did not, we used the Mann-Whitney U test to compare GMC between the two groups.

We also analyzed post-vaccination GMCs in participants who were seropositive and seronegative at baseline. We used Kolmogorov-Smirnov test of normality to check distribution of antibody levels. Since they are not normal, we used Kruskal-Wallis test to make comparisons inside each group.

Logistic regression was used to identify variables of interest associated with seroconversion among SOT participants, including demographic characteristics, immunosuppressive drugs used and the type of organ transplanted. First, univariate analysis was performed, using Wilcoxon test or Qui-square or Fischer Exact Test. Variables with a significance level of 0.20 were selected to the multivariate analysis.

Adverse events were described according to frequency and intensity/grade. Comparison of frequency of solicited AE between SOT and immunocompetent groups was performed using the chi-square test.

All participants were enrolled only after having all questions answered, reading and signing an informed consent form. The study was approved by the Ethics Committee for Analysis of Research Projects (CAPPesq) of the HC-FMUSP (CAAE 66795817.9.0000.0068).

## 3 Results

### 3.1 Demographic and clinical characteristics

**Figure 1:**
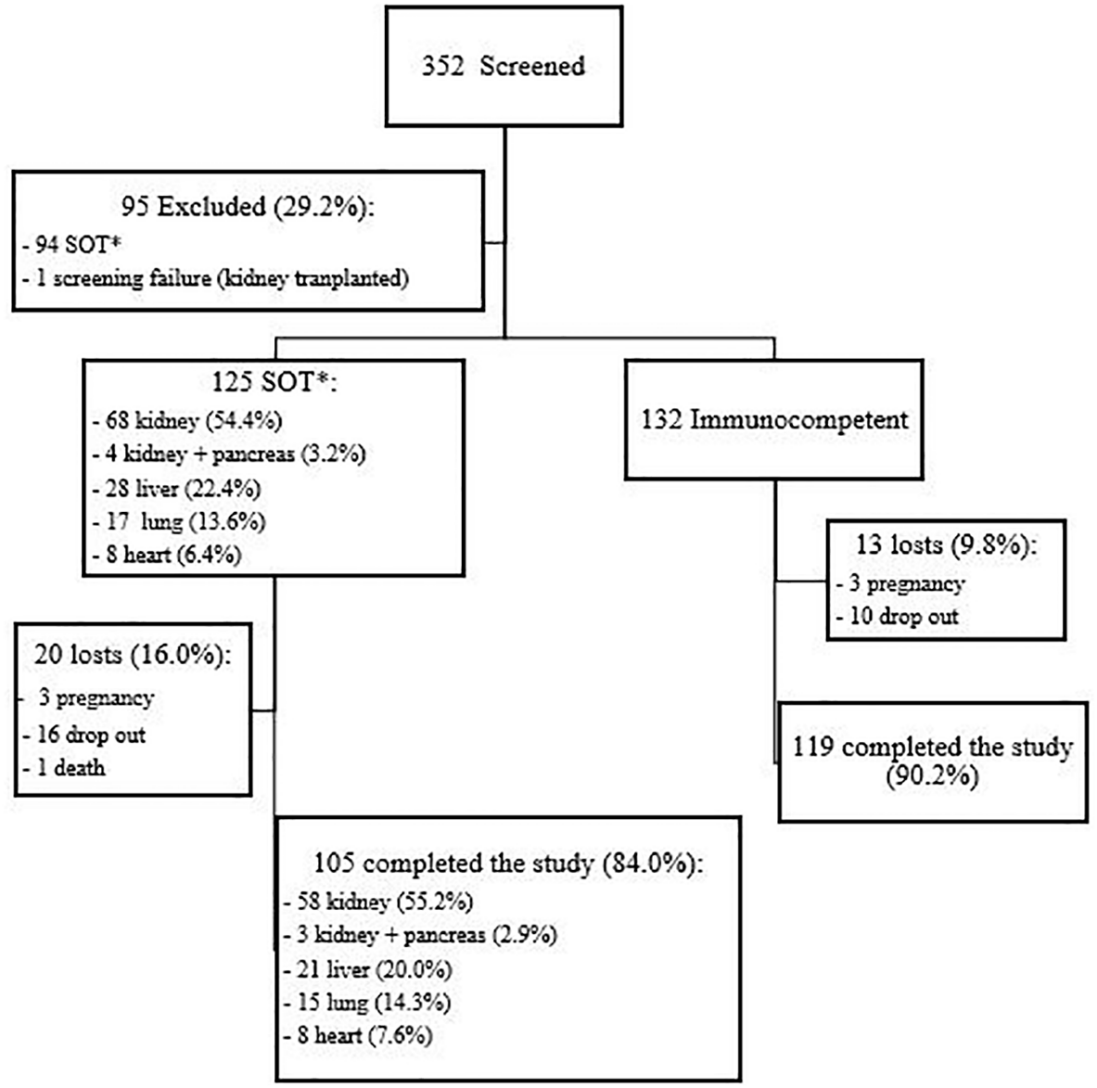
Flowchart of study population. *SOT, solid organ transplanted.

Overall, 257 women aged 18 to 45 years were enrolled: 125 SOT recipients (68 kidney, 4 kidney and pancreas, 28 liver, 17 lung and 8 heart) and 132 immunocompetent women. **Figure 1** shows the flowchart of participants screened, included and that completed the study. Kidney and pancreas transplant recipients were analyzed together with the kidney transplant recipients. **Table 1** shows the participants demographic and clinical characteristics. SOT recipients were a little older than immunocompetent women (35.0 and 32.5 years, respectively, *p*=0.002), had lower educational level (10.8 and 14.3 years of schooling, *p*<0.001) and used less contraception (72.8% among SOT vs 54.5% among immunocompetent women, *p*=0.002). As expected, comorbidities were more frequent in SOT recipients (69.6% vs 33.3%, *p*<0.001), particularly arterial hypertension, diabetes and dyslipidemia.

**Table 1:** Demographic and clinical characteristics of 257 participants included in study of the quadrivalent HPV vaccine (4vHPV) in female solid organ transplant recipients compared to immunocompetent women, Sao Paulo, Brazil, 2017-2019.

	Solid organ transplant recipients (N=125)	Immunocompetent (N=132)	*p-value*
Age (years)			
Mean (SD) Median (minimum – maximum)	35.0 (6.8) 37.0 (18.5 – 45.3)	32.5 (6.3) 33.0 (18.2 – 45.6)	**0.002**
Color, n (%)			
White Black Asian	64 (51.2) 58 (46.4) 3 (2.4)	74 (56.1) 52 (39.4) 6 (4.5)	0.394
Years of schooling			
Mean (SD) Median (minimum – maximum)	10.8 (3.6) 11.0 (0 - 17)	14.3 (3.0) 15.0 (3 - 22)	**<0.001**
Age at sexual debut (years)			
Mean (SD) Median (minimum – maximum)	18.2 (3.8) 18 (12 - 33)	18.5 (3.1) 18 (12 - 30)	0.443
Number of lifetime sexual partners, n (%)			
0 1 - 2 3 - 5 ≥6 Unknown	13 (10.4) 51 (40.8) 46 (36.8) 15 (13.0) 0	5 (3.8) 43 (32.6) 59 (44.7) 24 (18.1) 1 (0.8)	0.072*
Current sexual partnership, n (%)			
Heterosexual Women who have sex with women None	93 (75.0) 1 (0.8) 30 (24.2)	100 (76.3) 4 (3.1) 27 (20.6)	0.364
Number of pregnancies, n (%)			
0 1 2 ≥3	67 (54.9) 26 (21.3) 13 (10.7) 16 (13.1)	79 (60.3) 30 (22.9) 14 (10.7) 8 (6.2)	0.358
Current contraceptive use, n (%)			
None Hormonal Intrauterine device (IUD) Hormonal and IUD	91 (72.8) 30 (24.0) 3 (2.4) 1 (0.8)	72 (54.5) 51 (38.6) 0 9 (6.8)	**0.002^#^**
History of sexually transmitted infections, n (%)			
None Hepatitis B Genital herpes Unknown	121 (96.0) 1 (0.8) 1 (0.8) 2 (1.6)	128 (97.0) 0 2 (1.5) 2 (1.5)	0.697^#^
Smoking history, n (%)			
Never smoked Current smoker Ex-smoker	115 (92.0) 2 (1.6) 8 (6.4)	116 (87.9) 8 (6.1) 8 (6.1)	0.181
Comorbidities, n (%)			
Arterial hypertension Diabetes Hypothyroidism Dyslipidemia Other None	49 (39.2) 17 (13.6) 8 (6.4) 10 (8.0) 41 (32.8) 38 (30.4)	5 (3.8) 1 (0.8) 5 (3.8) 1 (0.8) 47 (35.6) 88 (66.7)	**<0.001**
Body mass index (kg/m^2^)			
Mean (SD) Median (minimum – maximum)	25.7 (4.8) 25.6 (15.1 – 39.2)	25.3 (4.8) 25.0 (17.2 – 39.9)	0.526
Interval from 1^st^ to 3^rd^ vaccine dose (days)			
Mean (SD) Median (minimum – maximum)	213 (42.6) 202 (179 – 384)	207 (60.4) 194 (181 - 672)	
Interval from 3^rd^ vaccine dose and blood drawn (days)			
Mean (SD) Median (minimum – maximum)	44.6 (17.6) 37 (21 – 106)	33.8 (8.6) 31 (24 – 86)	

*Comparison between groups, considering 1-2 vs >2 sexual partners.

^#^Comparison between groups, considering Yes vs No response.

Bold values = statistically significant.

In the SOT group, mean time since transplantation was 5.6 years (SD 4.9; median 4.0 years). Most SOT recipients used immunosuppressive regimens including mycophenolate mofetil (MMF), corticosteroids and tacrolimus (52%), followed by azathioprine, corticosteroids and tacrolimus (11.2%) and MMF, corticosteroids and cyclosporine (8%).

### 3.2 Immune response e to 4vHPV vaccine

A total of 224 participants (105 [84.0%] SOT recipients and 119 [90.2%] immunocompetent women) completed the study, i.e., received three doses of the 4vHPV vaccine and collected blood samples at baseline and after the third vaccine dose. There were 33 dropouts: 20 SOT recipients (3 pregnancies, 16 lost-to-follow-up and 1 death) and 13 immunocompetent women (3 pregnancies and 10 lost-to-follow-up).

There were delays in completing the vaccination schedule, expected to be 180 days. The mean interval time to complete vaccination was 213 days (SD 42.6) in the SOT group and 207 days (SD 33.8) in the immunocompetent women group. There were also delays in collecting blood sample after completing vaccination, proposed to occur approximately 30 days after the third vaccine dose. The mean interval between completing vaccination and blood drawn was 44.6 days (SD 17.6) in the SOT group and 33.8 days (SD 8.6) in the immunocompetent group. Due to dropouts and delays in vaccine uptake and post-vaccination blood sample collection, it was not possible to perform a per-protocol analysis.

**Figure 2:**
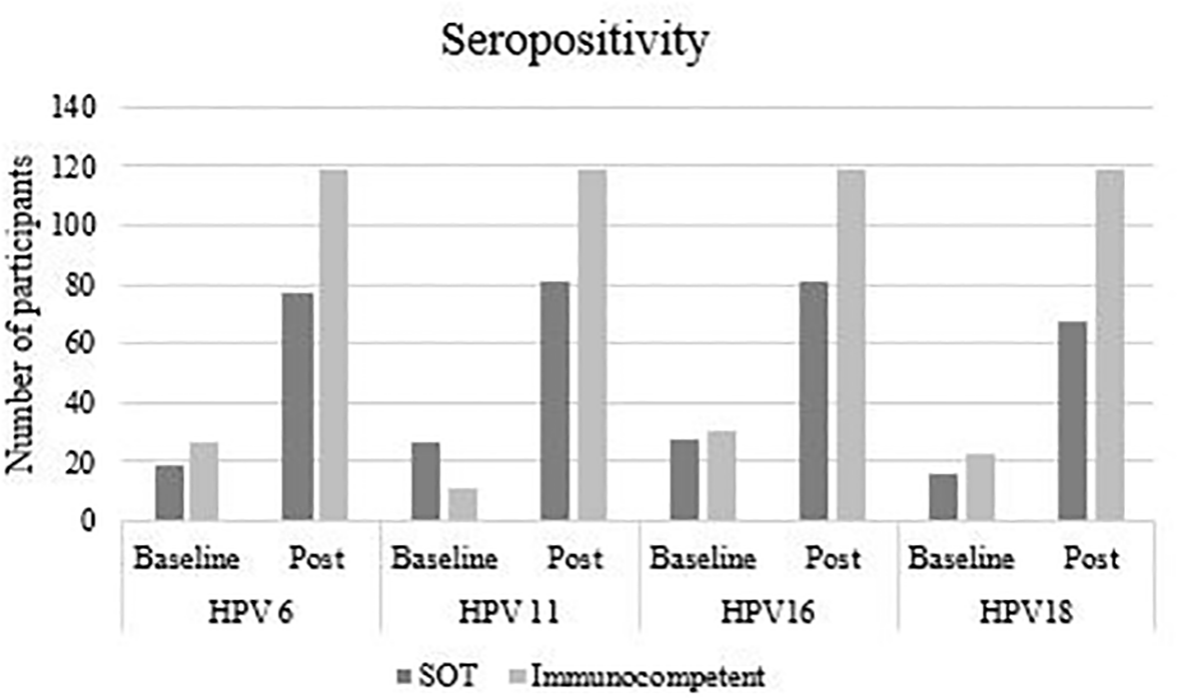
Baseline and post-vaccination seropositivity, according to HPV type and study group.

**Table 2** shows HPV-type specific seropositivity rates at baseline and after completing vaccination, seroconversion rates (from HPV type-specific seronegative at enrolment to seropositive post-vaccination) and antibody GMC, by study group. At baseline, 67 (63.8%) SOT recipients and 71 (59.7%) immunocompetent women were seropositive for at least one HPV type, most frequently to HPV16 (26.7% and 26.9% of SOT recipients and immunocompetent women, respectively), HPV11 (25.7% and 9.2%), HPV6 (18.1% and 23.5%) and HPV52 (22.9% and 19.3%, respectively). **Figure 2** shows baseline and post vaccination seropositivity for each 4vHPV type in both SOT and immunocompetent groups.

**Table 2:** HPV seropositivity rates at baseline and post-4vHPV vaccination; seroconversion rates and antibody geometric mean concentrations (GMC), according to HPV types, in solid organ transplant (SOT) recipients and immunocompetent (IC) women.

	Seropositivity rates at baseline		Seropositivity rates post-4vHPV vaccination		Seroconversion rates *				Antibody geometric mean concentrations (GMCs) IU/mL			
Group	SOT	IC	SOT	IC	SOT	IC			SOT		IC	
HPV type	(N=105) n (%)	(N=119) n (%)	(N=105) n (%)	(N=119) n (%)	n seropositive / N seronegative at enrolment (%)	n seropositive/ N seronegative at enrolment (%)	p**	n	GMC (95% CI)	n	GMC (95% CI)	p***
**6**	19 (18.1)	28 (23.5)	77 (73.3)	119 (100)	59 / 86 (68.6)	91/ 91 (100)	**<0.0001**	59	7.18 (3.97 - 12.96)	91	82.71 (62.05 - 110.26)	**<0.001**
**11**	27 (25.7)	11 (9.2)	81 (77.1)	119 (100)	56 / 78 (71.8)	108 / 108 (100)	**<0.0001**	56	3.18 (1.78 - 5.68)	108	45.11 (36.18 - 56.24)	**<0.001**
**16**	28 (26.7)	32 (26.9)	81 (77.1)	119 (100)	53 / 77 (68.8)	87 / 87 (100)	**<0.0001**	53	44.19 (21.24 - 91.96)	87	401.16 (315.6 - 509.85)	**<0.001**
**18**	16 (15.2)	24 (20.2)	67 (63.8)	119 (100)	51 / 89 (57.3)	95 / 95 (100)	**<0.0001**	51	14.76 (7.00 - 31.12)	95	116.81 (87.47 - 155.97)	**<0.001**
**31**	9 (8.6)	13 (10.9)	18 (17.1)	62 (52.1)	11 / 96 (11.4)	49 / 106 (46.2)	**<0.0001**	11	1.49 (0.74 - 3.00)	49	1.19 (0.76 - 1.87)	0.473
**33**	11 (10.5)	12 (10.1)	29 (27.6)	78 (65.6)	18 / 94 (19.2)	67 / 107 (62.6)	**<0.0001**	18	0.31 (0.15 - 0.63)	67	0.25 (0.18 - 0.35)	0.461
**52**	24 (22.9)	23 (19.3)	30 (28.6)	39 (32.8)	12 / 81 (14.8)	17 / 96 (17.7)	0.6857	12	0.72 (0.42 - 1.25)	17	1.91 (0.91 - 4.02)	0.117
**58**	17 (16.2)	15 (12.6)	28 (26.7)	52 (43.7)	12 / 88 (13.6)	39 / 104 (37.5)	0.0003	12	0.21 (0.12 - 0.36)	39	0.27 (0.16 - 0.43)	0.921

SOT, solid organ transplant. *Seroconversion analyses: from type-specific seronegative at enrolment to seropositive post-vaccination. **Chi-square test. ***Mann-Whitney U t-test.

Sao Paulo, Brazil, 2017-2019.

Bold values = statistically significant.

**Table 3** shows the antibody GMC of each type HPV of participants seropositive and seronegative at the baseline in both SOT and immunocompetent groups. Post-vaccination antibody levels of HPV6 and 16 were significantly higher in SOT participants who were seropositive at baseline in comparison to SOT who were seronegative.

**Table 3:** Post- vaccination anti-HPV antibody geometric mean concentrations (GMC) in participants who were seropositive and seronegative at baseline, according to group (solid organ transplant [SOT] recipients and immunocompetent women) AND hpv TYPE.

HPV type	Seropositive at baseline		Soronegative at baseline		p*
	n	GMC IU/mL (IC 95%)	n	GMC IU/mL (IC 95%)	
Solid organ transplanted					
HPV 6	18	101 (36.8 – 277.3)	77	13.3 (7.6 – 23.3)	<0.00001
HPV 11	25	8.6 (2.9 – 25)	81	4.3 (2.6 – 7.2)	0.203
HPV 16	28	118.9 (48.9 – 288.7)	81	62.2 (35.3 – 109.7)	0.034
HPV 18	16	34.1 (10.1 – 115.2)	67	18 (9.6 – 33.8)	0.295
Immunocompetent					
HPV 6	27	109 (62.4 – 192.7)	92	123.7 (70.8 – 204.6)	0.936
HPV 11	11	46.7 (23.5 – 92.9)	108	47.5 (37.6 – 60.1)	0.211
HPV 16	31	383.2 (267.4 – 549)	88	470 (366.6 – 602.7)	0.198
HPV 18	23	224.3 (109.6 – 459)	96	135.9 (101.9 – 181.1)	0.378

*Kruskal-Wallis test.

Only participants seronegative for the specific HPV type at baseline were included in the seroconversion analyses. In the SOT group, 4vHPV vaccine types seroconversion rates were 57.3% (for HPV18), 68.6% (HPV16), 68.8% (HPV6) and 71.8% (HPV11), whereas 100% seroconversion for all vaccine types was observed in the immunocompetent group. The differences were statistically significant for all four vaccine types (*p*<0.0001). 4vHPV vaccine types antibody GMCs were also lower in the SOT group compared to the immunocompetent group, and the differences were statistically significant for all four vaccine types (*p*<0.0001).

As the estimated sample size was not reached, we estimated the pos-hoc power of the study to find a difference of 10% in seroconversion rates between the two groups for each HPV vaccine type (6, 11, 16 and 18), considering the number of seronegative participants at baseline and alpha 0.05, and found the power of the study was >80% for all four HPV types (**Table 4**)

**Table 4:** *Post-hoc* power of the study to detect a difference of 10% in seroconversion rates for each HPV vaccine type (6, 11, 16 and 18), considering the numbers of seronegative participants at baseline alpha 0.05.

HPV type	Seronegative participants (baseline)		Seroconversion (%)		*Post-hoc* power (%)
	SOT (n)	Immunocompetent (n)	SOT (n)	Immunocompetent (n)	
6	86	91	89.9	99.9	86.5
11	78	108	89.9	99.9	88.4
16	77	87	89.9	99.9	84.1
18	89	95	89.9	99.9	87.8

Both groups had seroconversion for HPV types included only in the 9vHPV vaccine. Seroconversion rates for HPV31, 33 and 58 were significantly lower in the SOT group than in immunocompetent. No statistically significant difference in seroconversion to HPV52 between the two groups was observed. Antibody GMCs for these HPV types were considerably lower than GMC of 4vHPV vaccine types without statistically significant differences between the two groups.

Only SOT women who were seronegative for all 4vHPV vaccine types at baseline (n=38) were included in the analyses of risk factors associated with lack of seroconversion for 4vHPV types. The outcome was seroconversion for at least three 4vHPV vaccine types. Univariate analysis considered: age, ethnicity, education, smoking, alcohol consumption, number of partners, transplanted organ, time since transplant, comorbidities, body mass index (BMI), leukopenia, lymphopenia and current immunosuppressive drugs (use of the MMF + corticosteroid + tacrolimus drug regimen or not, use of one or two immunosuppressive drugs or use of three or four immunosuppressive drugs, use of the MMF drug or not, use of cyclosporine and/or tacrolimus or not and use of sirolimus and/or everolimus or not. In the univariate analysis, the type of transplanted organ, dyslipidemia, leukopenia, MMF use, MMF + corticosteroid + Tacrolimus and use of three or four immunosuppressive drugs had a significance of ≤0.20 and were included in the multivariate analysis. The multiple logistic regression showed that kidney transplant (aOR=0.11, 95%CI 0.01-0.78; *p*=0.056) and heart transplant (aOR=0.08, 95%CI 0-0.78; *p*=0.07) were associated to lower seroconversion rates as compared to liver transplant. The immunosuppressive regimen (MMF, corticosteroids and tacrolimus) was also associated with lower seroconversion rates (aOR=0,22, 95%CI 0.06-0.77; *p*=0.021) (**Table 5**).

**Table 5:** Multiple logistic regression to analyze risk factors associated with lack of seroconversion for 4vHPV types among SOT recipients.

Variable	Estimation	Standard error	z-value	*p*-value	Odds ratio	Confidence interval 95%
Heart tranplantation*	-2.56	1.42	-1.81	0.070	0.08	0.00 – 0.98
Lung transplantation*	-1.17	1.32	-0.89	0.374	0.31	0.01 – 3.52
Kidney transplantation*	-2.20	1.15	-1.91	0.056	0.11	0.01 – 0.78
MMF + corticosteroid + Tacrolimus: Yes**	-1.51	0.65	-2.30	0.021	0.22	0.06 – 0.77

*Compared to liver transplantation.

**Compared to use of any other immunosuppressive drug combination.

### 3.3 4vHPV vaccine safety

**Figure 3:**
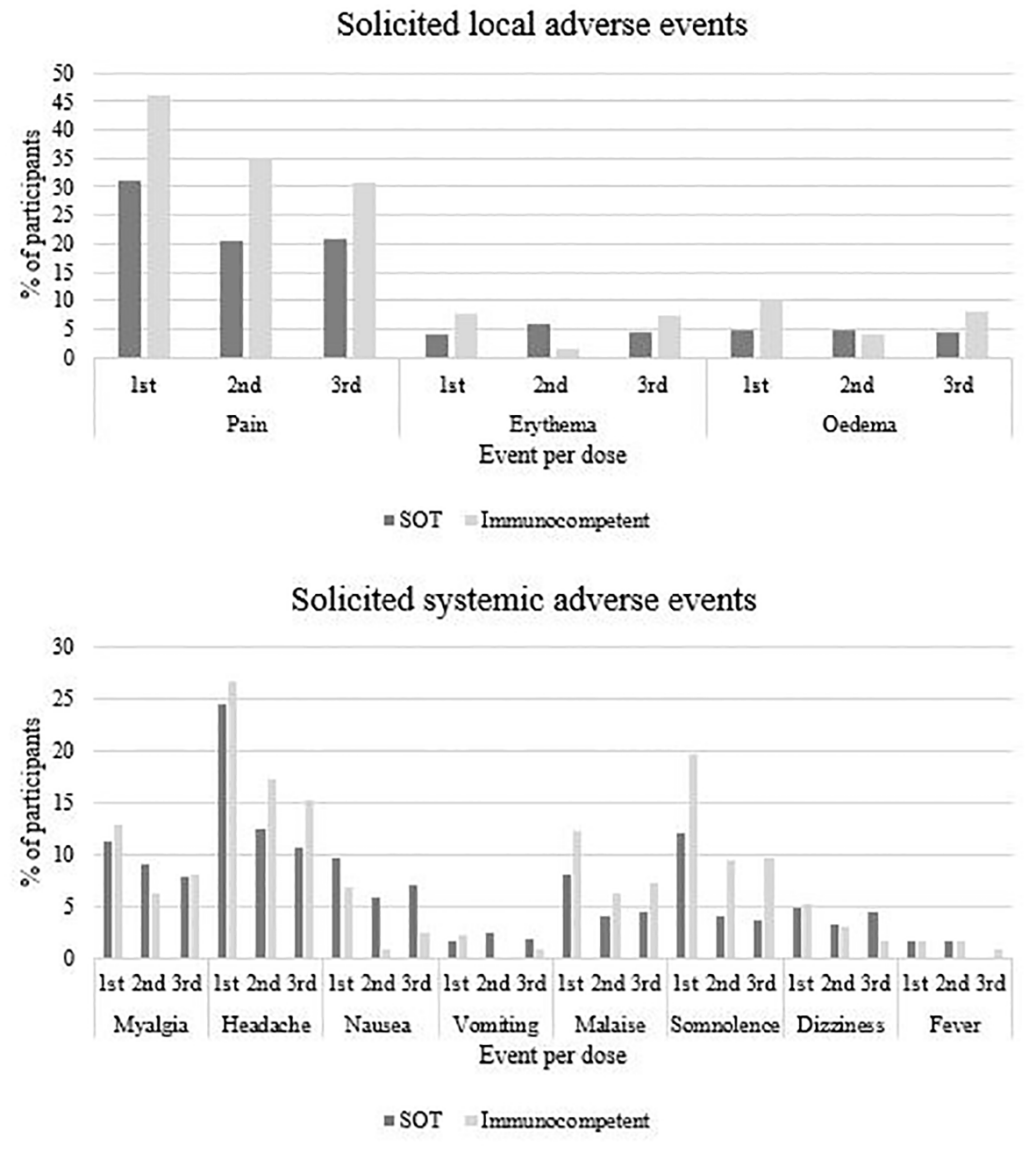
Solicited local and systemic adverse events following quadrivalent HPV vaccination according and study group and vaccine doses.

Three participants (two SOT and one immunocompetent) had lipothymia within 30 minutes after 4vHPV vaccination, but did not request medical care. **Table 6** shows solicited adverse events that occurred within seven days after each vaccine dose, according to intensity and by study group. **Table 7** shows frequency of unsolicited adverse events in both solid organ transplant (SOT) recipients and immunocompetent groups according to HPV4v dose. **Figure 3** shows proportions of participants with solicited local and unsolicited adverse events in both groups. Pain in the injection site and headache were the most frequent AE in both groups. Local pain was more frequent in immunocompetent women than in SOT recipients following all three doses. Other AEs rates were comparable in both groups. Most AEs were mild. Six events were classified as intensity grade 4 because the participants sought medical attention, but none required hospitalization.

**Table 6:** Solicited adverse events in the seven days after each 4vHPV vaccine dose, classified by intensity grade, in 125 solid organ transplant (SOT) recipients and 132 immunocompetent women, Sao Paulo, Brazil, 2017-2019.

Solicited adverse events	SOT						Immunocompetent						
	Grade 1	Grade 2	Grade 3	Grade 4	No	Unknown	Grade 1	Grade 2	Grade 3	Grade 4	No	Unknown	p*
	n (%)	n (%)	n (%)	n (%)	n (%)	n (%)	n (%)	n (%)	n (%)	n (%)	n (%)	n (%)	
Local pain													
1^st^ dose	34 (27.2)	3 (2.4)	2 (1.6)	0	81 (64.8)	5 (4.0)	51 (38.6)	6 (4.7)	1 (0.8)	0	71 (53.8)	3 (2.3)	**0.044**
2^nd^ dose	24 (19.8)	1 (0.8)	0	0	79 (63.2)	17 (14.0)	36 (27.3)	10 (7.9)	0	0	71 (55.9)	10 (7.9)	**0.015**
3^rd^ dose	21 (18.6)	3 (2.4)	0	0	72 (57.6)	17 (15.0)	34 (27.4)	4 (3.2)	0	0	50 (10.3)	36 (29.0)	**0.009**
Local erythema													
1^st^ dose	3 (2.4)	2 (1.6)	0	0	115 (92.0)	5 (4.0)	7 (5.3)	3 (2.3)	0	0	119 (90.2)	3 (2.3)	0.235
2^nd^ dose	7 (5.8)	0	0	0	97 (80.2)	17 (14.0)	1 (0.8)	1 (0.8)	0	0	115 (90.6)	10 (7.9)	0.059
3^rd^ dose	5 (4.4)	0	0	0	91 (80.5)	17 (15.0)	5 (4.0)	1 (0.8)	0	0	82 (66.1)	36 (29.0)	0.645
Local oedema													
1^st^ dose	4 (3.2)	2 (1.6)	0	0	114 (91.2)	5 (4.0)	10 (7.6)	2 (1.5)	0	0	117 (88.6)	3 (2.3)	0.19
2^nd^ dose	5 (4.1)	1 (0.8)	0	0	98 (81.0)	17 (14.0)	3 (2.4)	2 (1.6)	0	0	112 (88.2)	10 (7.9)	0.61
3^rd^ dose	4 (3.5)	1 (0.9)	0	0	91 (80.5)	17 (15.0)	5 (4.0)	5 (4.0)	0	0	82 (66.1)	36 (29.0)	0.152
Myalgia													
1^st^ dose	11 (8.8)	1 (0.8)	1 (0.8)	1 (0.8)	106 (84.8)	5 (4.0)	14 (10.6)	1 (0.8)	2 (1.5)	0	112 (84.8)	3 (2.3)	0.718
2^nd^ dose	6 (5.0)	3 (2.5)	1 (0.8)	1 (0.8)	93 (76.9)	17 (14.0)	5 (3.9)	3 (2.4)	0	0	109 (85.8)	10 (7.9)	0.322
3^rd^ dose	4 (3.5)	4 (3.5)	1 (0.9)	0	87 (77.0)	17 (15.0)	5 (4.0)	5 (4.0)	0	0	78 (62.9)	36 (29.0)	0.658
Headache													
1^st^ dose	22 (17.6)	4 (3.2)	3 (2.4)	1 (0.8)	90 (72.0)	5 (4.0)	20 (15.2)	10 (7.6)	5 (3.8)	0	90 (71.2)	3 (2.3)	0.283
2^nd^ dose	4 (3.3)	8 (6.6)	1 (0.8)	2 (1.7)	89 (73.6)	17 (14.0)	17 (13.4)	5 (3.9)	0	0	95 (74.8)	10 (7.9)	0.384
3^rd^ dose	6 (5.3)	3 (2.7)	3 (2.7)	0	84 (74.3)	17 (15.0)	11 (8.9)	6 (4.8)	2 (1.6)	0	69 (55.6)	36 (29.0)	0.100
Nausea													
1^st^ dose	10 (8.0)	1 (0.8)	1 (0.8)	0	108 (86.4)	5 (4.0)	5 (3.8)	3 (2.3)	1 (0.8)	0	120 (90.9)	3 (2.3)	0.391
2^nd^ dose	5 (4.1)	2 (1.7)	0	0	97 (80.2)	17 (14.0)	1 (0.8)	0	0	0	116 (91.3)	10 (7.9)	**0.020**
3^rd^ dose	4 (3.5)	2 (1.8)	2 (1.8)	0	88 (77.9)	17 (15.0)	1 (0.8)	2 (1.6)	0	0	85 (68.5)	36 (29.0)	0.160
Vomiting													
1^st^ dose	1 (0.8)	0	1 (0.8)	0	118 (94.4)	5 (4.0)	0	2 (1.5)	1 (0.8)	0	126 (95.5)	3 (2.3)	0.137
2^nd^ dose	2 (1.7)	0	0	1 (0.8)	101 (83.5)	17 (14.0)	0	0	0	0	117 (92.1)	10 (7.9)	0.103
3^rd^ dose	0	0	2 (1.8)	0	94 (83.2)	17 (15.0)	0	1 (0.8)	0	0	87 (70.2)	36 (29.0)	0.612
Malaise													
1^st^ dose	8 (6.4)	0	2 (1.6)	0	110 (88.0)	5 (4.0)	10 (7.6)	4 (3.1)	2 (1.5)	0	112 (85.5)	3 (2.3)	0.284
2^nd^ dose	1 (0.8)	4 (3.3)	0	0	98 (81.0)	17 (14.0)	5 (3.9)	2 (1.6)	1 (0.8)	0	109 (85.8)	10 (7.9)	0.534
3^rd^ dose	1 (0.9)	3 (2.7)	1 (0.9)	0	91 (80.5)	17 (15.0)	3 (2.4)	6 (4.8)	0	0	79 (63.7)	36 (29.0)	0.120
Somnolence													
1^st^ dose	13 (10.5)	1 (0.8)	1 (0.8)	0	104 (83.9)	6 (4.8)	21 (15.9)	3 (2.3)	2 (1.5)	0	103 (78.0)	3 (2.3)	0.110
2^nd^ dose	4 (3.3)	1 (0.8)	0	0	99 (81.8)	17 (14.0)	10 (7.9)	2 (1.6)	0	0	105 (82.7)	10 (7.9)	0.129
3^rd^ dose	1 (0.9)	0	3 (2.7)	0	91 (81.2)	17 (15.0)	6 (4.8)	5 (4.0)	1 (0.8)	0	76 (61.3)	36 (29.0)	0.800
Dizziness													
1^st^ dose	6 (4.8)	0	0	0	114 (91.2)	5 (4.0)	4 (3.0)	1 (0.8)	2 (1.5)	0	122 (92.4)	3 (2.3)	0.880
2^nd^ dose	2 (1.7)	1 (0.8)	1 (0.8)	0	100 (82.6)	17 (14.0)	0	4 (3.1)	0	0	113 (89.0)	10 (7.9)	0.865
3^rd^ dose	3 (2.7)	0	2 (1.8)	0	91 (80.5)	17 (15.0)	2 (1.6)	0	0	0	86 (69.4)	36 (29.0)	0.300
Fever ^#^													
1^st^ dose	1 (0.8)	1 (0.8)	0	0	117 (93.6)	6 (4.8)	1 (0.8)	0	1 (0.8)	0	114 (87.0)	15 (11.5)	0.979
2^nd^ dose	2 (1.6)	0	0	0	101 (84.2)	17 (14.2)	2 (1.6)	0	0	0	115 (90.6)	10 (7.9)	0.897
3^rd^ dose	0	0	0	0	96 (85.0)	17 (15.0)	1 (0.8)	0	0	0	87 (70.2)	36 (29.0)	0.478

*Statistical analysis using Chi-square or Fisher’s exact test comparing two groups considering whether or not they had the adverse event.

^#^One immunocompetent and one transplanted referred fever but did not measure temperature.

Bold values = statistically significant.

**Table 7:** Frequency of unsolicited adverse events in both solid organ transplant (SOT) recipients and immunocompetent groups according to HPV4v dose.

Event	1^st^ dose		2^nd^ dose		3^rd^ dose	
	SOT n (%)	Immunocompetent n (%)	SOT n (%)	Immunocompetent n (%)	SOT n (%)	Immunocompetent n (%)
Altered menstruation	3 (6.3)	5 (13.9)	1 (5.3)	0	0	0
Lip or eyelid oedema	2 (4.2)	1 (2.8)	1 (5.3)	1 (5.0)	1 (5.6)	3 (25.0)
Artralgia	1 (2.1)	0	1 (5.3)	0	1 (5.6)	0
Malaise	1 (2.1)	2 (5.6)	0	0	0	0
Abdominal pain	2 (4.2)	2 (5.6)	3 (15.8)	1 (5.0)	0	0
Menstrual cramps	2 (4.2)	0	0	1 (5.0)	0	0
Diarrhea	6 (12.5)	6 (16.7)	3 (15.8)	11 (55.0)	7 (38.9)	3 (25.0)
Equimosis at administration site	2 (4.2)	1 (2.8)	0	1 (5.0)	1 (5.6)	1 (8.3)
Exanthema	2 (4.2)	3 (8.3)	1 (5.3)	1 (5.0)	1 (5.6)	2 (16.7)
Flu-like symptoms	10 (20.8)	2 (5.6)	3 (15.8)	1 (5.0)	1 (5.6)	0
Lipothymia	1 (2.1)	1 (2.8)	1 (5.3)	0	0	0
Diffuse pruritus	1 (2.1)	2 (5.6)	1 (5.3)	0	2 (11.1)	0
Heat at administration site	0	0	0	0	1 (5.6)	0
Pruritus	3 (6.3)	1 (2.8)	1 (5.3)	0	1 (5.6)	0
Wheezing	1 (2.1)	0	2 (10.5)	0	2 (11.1)	1 (8.3)
Other	11 (22.9)	10 (27.8)	1 (5.3)	3 (15.0)	0	2 (16.7)
Total	48 (100.0)	36 (100.0)	19 (100.0)	20 (100.0)	18 (100.0)	12 (100.0)

There were two serious adverse events, one lung transplanted woman died from chronic organ transplant rejection due to non-adherence to the immunosuppressive treatment. She had received just the first vaccine dose and the death was considered unrelated to the vaccine. One liver transplanted participant had an episode of rejection after the first vaccine dose and her assistant physician contraindicated the subsequent vaccine doses. She was lost to follow up. We could not get enough data to evaluate the association of the episode with the vaccine.

## 4 Discussion

### 4.1 Immune response

The present study evaluated the immunogenicity and safety of the 4vHPV vaccine among solid organ transplanted women aged 18 to 45 years compared to immunocompetent women of the same age. We found very strong evidence of lower seroconversion rates (from 57% for HPV18 to 72% for HPV11) in SOT recipients compared to immunocompetent women (100% for all four vaccine types, *p*<0.001) and lower anti-HPV GMCs for all four vaccine types in SOT recipients (*p*<0.001).

Similar results in SOT recipients were found by other authors. A phase 3 trial carried out in Belgium evaluated the immunogenicity and safety of the nonavalent (9v) HPV vaccine in 171 SOT (kidney, heart or lung) recipients (women and men) and 100 persons living with HIV/AIDS (PLHA) aged 18 to 55 years (Boey et al., 2021). HPV antibody was measured by competitive Luminex immunoassay (cLIA). The mean age of the SOT recipients was higher (46.7 years) than in our study (35 years) and most participants were male (69%). They found 100% seroconversion rates to all nine vaccine types among PLHA; SOT recipients had significantly lower seroconversion rates and GMTs than PLHA. Seroconversion rates for the 4vHPV4v vaccine types in SOT recipients (HPV6, 64.3%; HPV11, 70.7%; HPV16, 69.1%; HPV18, 51.7%) were similar to our findings (Boey et al., 2021).

Another study evaluated the 4vHPV vaccine immunogenicity and tolerability in 29 male and female kidney transplant recipients aged from 9 to 18 years from seven centers in the United States (Nailescu et al., 2020). Seroconversion rates after vaccination were also similar to those observed in our study for HPV6 (72.4% vs. 69% in our study), HPV11 (69% vs. 72% in our study) and HPV18 (62.1% vs. 57% in our study), but were higher for HPV16 (89.7% vs. 69% in our study). GMCs were not comparable to our results, due to the different laboratory methodology (cLIA was used in the North American study) (Nailescu et al., 2020).

Three other smaller studies also evaluated the 4vHPV vaccine immunogenicity in SOT recipients. A Canadian study included 38 SOT men and women, aged 18 to 35 years, with no history of HPV-related diseases (Kumar et al., 2013). The participants were younger than in our study (median age, 25.9 and 35 years, respectively). The study used two methods to measure anti-HPV antibodies, HPV4-plex ELISA IgG and cLIA, obtaining different results according to test. ELISA seroconversion rates (HPV6 63.2%; HPV11 68.4%; HPV16 63.2% and HPV18 52.6%) were similar to those found in our study (69%; 72%; 69% and 57%, respectively). cLIA seroconversion rates for HPV11 (66.7%) and HPV16 (51.9%) were also similar, whereas seroconversion for HPV6 (23.1%) and HPV18 (14.8%) were lower than by ELISA and compared to our findings (Kumar et al., 2013).

A study conducted in USA included kidney or liver transplant recipients of both sexes, aged from nine to 17 years (Gomez-Lobo et al., 2014). However, this study was interrupted due to an increase in cases of acute rejection in kidney transplant recipients, and so only eight participants (7 kidney and one liver) completed the study and were analyzed. The results were unexpected and different from our findings and all other published studies, as all eight participants seroconverted for all four HPV vaccine types. Six (42.8%) participants developed acute rejection in a mean of 3.6 months after vaccination. However, the association of the rejection episodes with vaccination was difficult to stablish, due to lack of information on participants’ adherence to immunosuppressive treatment. In our study, there was two episodes of draft rejection. One in a lung transplanted woman without adherence to the immunosuppressive therapy and another in a liver transplanted participant that dropped out of the study and so we did not have enough data to analyze the association of the episode with HPV vaccination (Gomez-Lobo et al., 2014).

Finally, another USA study evaluated 23 kidney transplant women, aged 9 to 21 years, who received the 4vHPV vaccine, compared to women of the same age at different stages of chronic kidney disease (Nelson et al., 2016). Blood samples for serology were collected from one to 12 months and from 12 to 35 months after completing the three-dose vaccine schedule, and HPV antibodies were measured by cLIA. After vaccination, seroconversion for HPV6 (63.6%) among kidney transplant recipients was similar to our study; HPV11 (63.7%) was slightly lower and for types 16 (100%) and 18 (72.7%) were higher. Among women with chronic kidney disease, 100% seroconversion for all four types were seen (Nelson et al., 2016).

In our study, seroconversion to additional alpha-HPV types included in the 9vHPV vaccine (HPV31, 33 and 58) was observed, mainly in the immunocompetent group. We cannot exclude participants’ exposure to these HPV types during the study period, but evidence of cross-reactivity for vaccine-related HPV types (HPV16-related: 31, 33, 35, 52, 58 and HPV18-related: 39, 45, 59, 68) has been previously reported (Malagón et al., 2012). Cross-protection seems to be more robust with the bivalent HPV (HPV16 and 18), but does also occur with 4vHPV vaccination (Malagón et al., 2012). A systematic review and meta-analysis (Malagón et al., 2012) including clinical trials that evaluated vaccine efficacy against HPV types not included in the bivalent and quadrivalent HPV vaccines showed 4vHPV vaccine efficacy against persistent infection and CIN-2 associated to HPV33, but no cross protection was found for HPV52 and HPV58 in studies with bi- or quadrivalent HPV vaccines. In the follow-up, antibody titers against HPV types not included in the vaccines dropped, which may suggest a decline in long-term cross-protection. On the other hand, antibody titers for vaccine types 16 and 18 persisted. More studies are needed to better evaluate cross-protection to vaccine-related HPV types in different population groups.

The role of HPV vaccination after treatment of HPV-related diseases have been raised (Bogani et al., 2024). Protection against recurrence of precancerous lesions may be another important contribution of HPV vaccination, but there is no consensus in this matter and more studies are needed to strongly support this indication of vaccination, particularly in immunocompromised hosts (Kechagias et al., 2022).

### 4.2 Safety

Regarding 4vHPV vaccine safety, our findings were similar to other studies involving SOT recipients (Kumar et al., 2013; Nelson et al., 2016; Nailescu et al., 2020). Local pain and systemic symptoms, such as headache are the most frequent findings, generally mild and self-limited. Local pain after each dose was more frequent in immunocompetent, which may be due to greater pain tolerance of SOT recipient women, or greater inflammation in the immunocompetent. Rejection was identified in two participants in our study, one considered not related to vaccination and the other was unclassified due to lack of information. In a previous study involving kidney transplant recipients (Nelson et al., 2016), few adverse events were identified after vaccination (11 cases in 23 participants, eight of which were pain at the injection site). Two transplant recipients had acute rejection after vaccination, with a rate (8.6%) similar to that reported annually in the US, which does not suggest an increased risk in this cohort (19).

### 4.3 Limitations and strengths

Our study had some limitations. First, the inclusion of women who self-reported lack of previous HPV-related lesions, without any request to show test results, may have led to some misclassification and enrolment of already infected women. HPV previous infection may have been omitted to get the vaccine free of charge in the study, since at the time of our study enrollment, the 4vHPV vaccine was available through the Brazilian health system (SUS or Unified Health System) only for SOT recipients up to 26 years of age and immunocompetent persons under 15 years of age. Second, we opted for not including men and so the results cannot be generalized to the male SOT recipients. Thirdly, losses to follow-up in our study (16% in the SOT group and 9.8% in the immunocompetent group) were important. One of the reasons for losses in the SOT group was the difficulty of transportation to attend the visits, since the protocol required four visits to the clinic, with relatively short intervals between them, shorter than the regular intervals between routine follow-up visits of stable SOT recipients. Our study had a number of strengths such as a good sample size, which is larger than most studies involving SOT recipients previously published and had >80% power to detect a 10% difference in seroconversion rates between the two groups. Finally, the inclusion of women with different types of SOT (kidney, kidney and pancreas, liver, heart and lung) and inclusion of a comparator group of immunocompetent women.

### 4.4 Conclusions

In conclusion, we found that the 4vHPV vaccine was safe in SOT women aged 18 to 45 years, but we found strong evidence of lower seroconversion rates and lower GMCs in SOT recipients compared to immunocompetent women of the same age. It is difficult to interpret these results, since correlates of protection had not been established, but they might suggest lower effectiveness. More studies on vaccine efficacy/effectiveness, duration of protection and alternative vaccination schedules to enhance immune response in this population are needed. Our results underscore the need to keep routine cervical cancer screening in SOT recipients regardless of HPV vaccination.

## Data Availability

The raw data supporting the conclusions of this article will be made available by the authors, without undue reservation.

## Study Group on HPV vaccine in immunosuppressed persons

Marta Heloisa Lopes, Edson Abdala, Paula Keiko Sato, Ligia Camera Pierrotti, Toni Ricardo Martins, Luiz Augusto Carneiro D’Albuquerque, William Carlos Nahas, Fernando Bacal, Tania Mara Varejao Strabelli, Ricardo Henrique de Oliveira Braga Teixeira, Silvia Vidal Campos, Carmen Diva Saldiva de André, Lúcia Pereira, Bruna Satie Umino, Raul Ossada, Camila Cristina Martini Rodrigues
